# Sam50 exerts neuroprotection by maintaining the mitochondrial structure during experimental cerebral ischemia/reperfusion injury in rats

**DOI:** 10.1111/cns.13967

**Published:** 2022-09-08

**Authors:** Xulong Yin, Jiahe Wang, Siyuan Yang, Haiying Li, Haitao Shen, Hui Wang, Xiang Li, Gang Chen

**Affiliations:** ^1^ Department of Neurology The First Affiliated Hospital of Soochow University Suzhou China; ^2^ Institute of Stroke Research Soochow University Suzhou China; ^3^ Department of Neurosurgery & Brain and Nerve Research Laboratory The First Affiliated Hospital of Soochow University Suzhou China

**Keywords:** ischemia–reperfusion injury, mitochondria, Sam50, Sam50–Mic19–Mic60 axis

## Abstract

**Aim:**

To investigate the role of Sam50, a barrel protein on the surface of the mitochondrial outer membrane, in cerebral ischemia–reperfusion (I/R) injury and its underlying mechanisms.

**Methods:**

A middle cerebral artery occlusion/reperfusion (MCAO/R) model in adult male Sprague–Dawley rats was established in vivo, and cultured neurons were exposed to oxygen–glucose deprivation/reoxygenation (OGD/R) to simulate I/R injury in vitro. Lentiviral vector encoding Sam50 or Sam50 shRNA was constructed and administered to rats by intracerebroventricular injection to overexpress and knockdown Sam50, respectively.

**Results:**

First, after MCAO/R induction, the mitochondrial structure was damaged, and Sam50 protein levels were increased responsively both in vivo and in vitro. Then, it was found that Sam50 overexpression could reduce infarction size, inhibit neuronal cell death, improve neurobehavioral disability, protect mitochondrial structure integrity, and ameliorate mitochondrial dysfunction, which was induced by I/R injury both in vivo and in vitro. However, Sam50 downregulation showed the opposite results and aggravated I/R injury by inducing neuronal cell death, neurobehavioral disability, and mitochondrial dysfunction. Moreover, we found that the interaction between Sam50 and Mic19 was broken off after OGD/R, showing that the Sam50–Mic19–Mic60 axis was breakage in neurons, which would be a reason for mitochondrial structure and function abnormalities induced by I/R injury.

**Conclusion:**

Sam50 played a vital role in the protection of neurons and mitochondria in cerebral I/R injury, which could be a novel target for mitochondrial protection and ameliorating I/R injury.

## Graphical abstract

Before I/R, Sam50, at the outer mitochondrial membrane, forms the Sam50–Mic19 axis with Mic19, which acts to stabilize the inner and outer mitochondrial membranes. When I/R occurs, Sam50–Mic19 axis fractures and the crista collapses. Overexpression of Sam50 can reversibly repair the broken axis, reduce mitochondrial damage, and improve the corresponding indicators, whereas knockdown of Sam50 led to the opposite result.

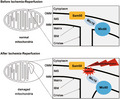

## 1 INTRODUCTION

At this stage, stroke has become the number one killer of human and disability in the world, and stroke is divided into ischemic stroke and hemorrhagic stroke.^1^, ^2^, ^3^ Ischemic strokes account for more than 80% of all strokes due to cerebral arterial embolism, and the most common is middle cerebral artery occlusion (MCAO).^2^, ^4^ When an ischemic stroke occurs, the death of neurons in the core infarction area is irreversible, but the penumbra area around the core infarction has the value of being saved.^5^ The study and exploration of nerve protection after ischemic stroke are still urgent.

Mitochondria is an important organelle that plays an essential role in cellular biological activities. Mitochondria are surrounded by two membranes: the outer mitochondrial membrane (OMM) and the inner mitochondrial membrane (IMM).^6^ The connections between mitochondrial intermembrane space bridging (IBM) and the cristae are the crista junctions (CJs), which are relatively uniform narrow, tubular, and slot‐like structures.^7^ Sam50 is an essential component of the sorting and assembly machinery (SAM) complex, which is essential for membrane integration and assembly of β‐barrel proteins into the mitochondrial outer membrane.^8^, ^9^ Mic19 and Mic 60, as the vital components of the mitochondrial contact site and cristae organizing system (MICOS) complex, play a key role in the structural stability of mitochondria.^10^, ^11^ Recent research shows that mic19 directly interacts with mitochondrial outer membrane protein Sam50 and inner membrane protein Mic60 to form the Sam50–Mic19–Mic60 axis, which dominantly connects SAM and MICOS complexes to assemble mitochondrial intermembrane space bridging (MIB) supercomplex for mediating mitochondrial outer and inner membrane contact.^12^

In this study, we aimed to explore the effects of Sam50 on mitochondrial dysfunction after ischemic stroke and its underlying mechanisms. A better understanding of the Sam50 function and mechanisms after I/R injury may provide insight into novel therapeutic targets for the treatment of stroke.

## 2 MATERIALS AND METHODS

### 2.1 Ethics and experimental animals

All the male adult Sprague–Dawley (SD) rats (250–300 g) were purchased from the Chinese Academy of Sciences. The standard of the animal housing conditions was a temperature of 25 ± 1°C and humidity of 50%–60%, with food and water available ad libitum. All the procedures were implemented strictly according to the ARRIVE Guidelines (Animal Research: Reporting of In Vivo Experiments) and controlled by the Animal Care and Use Committee of Soochow University for relieving the suffering of the animals. The animal data reporting has followed the ARRIVE 2.0 guidelines.^13^

### 2.2 Middle cerebral artery occlusion/reperfusion (MCAO/R) model

Rats were anesthetized with sevoflurane gas, and the temperature was kept at 37 ± 0.5°C with a heating lamp under the microscope operation. Then, the right common external and internal carotid artery (CCA, ECA, and ICA) were displayed through a midline neck incision. Next, we used a 4.0 monofilament nylon suture with a blunt tip polylysine coating to occlude the middle cerebral artery (MCA).^14^, ^15^ The nylon suture was inserted through the stump of the right ECA, from the ECA back into the ICA, until the tip occluded the proximal stem of the MCA. After that, MCA ischemia was kept for 2 h, which was a commonly accepted time, and then the nylon suture was pulled out to allow reperfusion.^16^, ^17^, ^18^ All the MCAO/R surgeries were performed by well‐trained and experienced researchers. Triphenyl tetrazolium chloride (TTC) staining of postoperative rat brain slices further showed that the establishment of the MCAO/R model in SD rats is mature and stable.

### 2.3 Measurement of cerebral blood flow

A laser speckle imaging system (RWD Life Science Co., Ltd.) was placed 10–15 cm above the closed cranial window. The skin and soft tissues on the cranial apical of the anesthetized rats are sufficiently separated. The saline solution was dropped on the closed cranial window, and the focus was adjusted to achieve a clear cerebral blood flow (CBF) image. The surface CBF images were captured before surgery, after ischemia, and after reperfusion. The photos were taken under the same zoom, gain, and pseudo‐color threshold settings.^19^

### 2.4 Intracerebral lentivirus injections

The SD rats were anesthetized with sevoflurane gas. After the anesthesia was successful, the rats were fixed on a stereotactic instrument. The skin was cut along the median sagittal line, and the muscle and periosteum were carefully separated to expose the skull. Three cortical injections of vector, LV‐Sam50, LV‐shRNA‐NC, and LV‐shRNA‐Sam50 were administered into the borehole in the right hemisphere with a stereotaxic instrument at the following coordinates from the bregma: point 1 at 1.0 mm anterior, 3.5 mm lateral, 2.5 mm deep; point 2 at −0.8 mm anterior, 3.5 mm lateral, 2.5 mm deep; and point 3 at −2.6 mm anterior, 3.5 mm lateral, 2.5 mm deep. Next, 10 ml of lentivirus (LV) suspension containing 9 × 10^8^ TU ml was injected at a rate of 0.5 ml/min. To avoid reverse flow, the needle was left in place for additional 5 min, withdrawn at a short distance, and then left in the new position for another 2 min before removal. At 5 days after injection, rats were euthanized with a lethal dosage of pentobarbital sodium, and the brains were removed for protein extraction and cryosection.^20^, ^21^ The transfection efficiency of LV was assessed by western blotting and immunofluorescence.

LV‐shRNA‐Sam50 sequences:

1. Sense: 5′‐GAGGAAGCGGAGTTTGTGGAA‐3′.
2. Sense: 5′‐ACGGACCAAGGATGACATCAT‐3′ (last choice).
3. Sense: 5′‐GAGCGTCCGAGGATTTAGCAT‐3′.

LV‐shRNA‐NC sequences:

Sense: 5′‐TTCTCCGAACGTGTCACGT‐3′.

### 2.5 Experimental groups and study design

*Part 1*: We analyzed the protein expression level of Sam50 after ischemia and reperfusion in in vivo experiment. A total of 54 rats (63 rats were used, 54 of which survived the operation) were randomly assigned to seven groups. The survival rate of rats is shown in Table S1. The number of sham and MCAO/R 6 h group was 12. A sham group and six experimental groups were arranged according to the following time points: 1, 3, 6, 12, 24, and 48 h after reperfusion. Six rats in each group were used for western blot analysis and electronic microscope sampling. And the other six rats in the sham and MCAO/R 6 h group were used for immunofluorescence analysis.

*Part 2*: Roles of Sam50 in I/R injury and the underlying mechanisms. In an in vivo experiment, 180 rats (200 rats were used), of which 180 rats survived the operation (the survival rate of rats is shown in Table S1) were arranged into six groups as follows: sham group, MCAO/R group, MCAO/R + Vector group, MCAO/R + LV‐Sam50 group, MCAO/R + LV‐shRNA‐NC group, and MCAO/R + LV‐shRNA‐Sam50 group. According to the results of Part 1, we performed western blot analysis and electron microscope sampling at 3 h after MCAO/R to determine the intervention effect (*n* = 6). Then, six rats per group were used for terminal deoxynucleotidyl transferase‐mediated dUTP nick end‐labeling (TUNEL) staining and Nissl staining. At 24 h after MCAO/R, six SD rats per group were used for TTC staining. Finally, 12 rats per group were used for neurobehavioral scores, adhesive removal test, and rotarod test after MCAO/R.

*Part 3*: Exploring the mechanism of action of Sam50 after oxygen–glucose deprivation/reoxygenation (OGD/R) in vitro. Cultured neurons were designed for six groups: control group, OGD/R group, OGD/R + Vector group, OGD/R + LV‐Sam50 group, OGD/R + LV‐shRNA‐NC group, and OGD/R + LV‐shRNA‐Sam50 group. Firstly, we explored the in vitro expression changes in Sam50 after the OGD/R model by western blot analysis and immunofluorescence analysis, and then the protective effect of Sam50 on neurons after oxygen and glucose deprivation was observed by live/dead cellular staining, JC‐1 staining, and Fluo‐4 AM staining. Finally, we dyed Sam50, Mic19, and DAPI together and co‐immunoprecipitation experiment to explore the connection between Sam50 and Mic19.

### 2.6 Western blot analysis

The rat penumbra cortex area was collected, removed, and placed in the lysis buffer containing phenylmethylsulfonyl fluoride (PMSF) for homogenization or used the lysis buffer to collect the nerve cells in the culture dish, and collected the supernatant from the homogenization into a 1.5 ml EP tube. The supernatant was centrifuged for 10 min at 12,000 *g* at 4°C. The protein in the supernatant was taken to determine its concentration by the BCA method. The proteins were then separated using SDS‐PAGE gel electrophoresis and transferred to the NC membrane. Then, it was blocked with 5% BSA for 1 h. After removing the membrane, the primary antibodies were incubated overnight and the NC membrane was washed three times with PBST (PBS + 0.1% Tween) the next day. After incubating the secondary antibody for 1 h, the NC membrane was washed three times with PBST, and then the enhanced probe was used to detect the blot chemiluminescence (ECL) (Millipore, United States) and the visualization imaging system (Bio‐Rad, United States).^22^, ^23^ Western blot data were passed through Image J software (National Institutes of Health, USA). For specific antibody information, please refer to Table S2.

### 2.7 Immunofluorescent microscopy

The brain samples were fixed with 4% paraformaldehyde and then embedded in paraffin and cut into 4 μm brain slices. The brain slices were placed in an oven at 70°C for 60 min and then deparaffinized with xylene and graded ethanol. After antigen retrieval with sodium citrate solution (MXB, China) and non‐specific‐binding blocking for 1 h, the brain slices were incubated with primary antibodies of Sam50, Mic19, and NeuN at 4°C overnight. Next, these brain slices were washed three times with PBST (PBS 0.1% + Tween 20) for 10 min each time, incubated with IgG antibodies for 1 h, and then these brain slices were washed with PBST (PBS 0.1% + Tween 20) for 10 min, three times, and covered with mounting medium containing 4,6‐diamino‐2‐phenylindole (DAPI; SouthernBiotech). Finally, a fluorescence microscope (Olympus BX50/BX‐FLA/DP70, Olympus Co.) was used to observe the brain slices for quantitative analysis.^24^

### 2.8 Electron microscopy

The rats were sacrificed within 1 min, and the brain tissue was fixed in 2.5% glutaraldehyde for 2–4 h. We rinsed the brain tissue three times with phosphoric acid rinsing solution, fixed with 1% osmium acid for 2 h at 4°C, rinsed three times with ddH2O, and then used ethanol gradient dehydration, propylene oxide transition, embedded after 812 resin gradient infiltration, and polymerization at 60°C. The embedded block was semi‐thin positioning and ultra‐thin sectioning with Leica UC7 ultra‐thin microtome, and the double‐stained section with uranyl acetate and lead citrate. Finally, we observed the ultrastructure of the tissue with a transmission electron microscope.

### 2.9 TUNEL staining

The brain tissue samples we obtained were embedded in paraffin and cut into 4 μm brain slices. The paraffin sections were dehydrated by heating in an oven at 70°C for 60–90 min and then deparaffinized with xylene and gradient ethanol. Then, put the components in Triton incubate in X‐100 for 10 min and washed three times with PBS. First, the stained slides were incubated with the TUNEL (Roche, Basel, Switzerland) reaction mixture at 37°C for 1 h, and then with NeuN (Abcam) overnight at 4°C. The next day, incubate with a suitable immunofluorescence secondary antibody at 37°C for 1 h, wash with PBS 3 times, and then cover it with DAPI. The fluorescence microscope was used to observe the TUNEL‐positive neurons in each section. Furthermore, we used Image J software to analyze the slices.^25^ The apoptotic index was defined as the average percentage of TUNEL‐positive cells in each section. The count of positive cells was statistically analyzed by an observer blinded to grouping.

### 2.10 Nissl staining

The brain slices were routinely deparaffinized and incubated with 0.5% toluidine blue at 55°C for 40 min, and then the stained brain slices were washed with graded ethanol and xylene. Finally, the neutral resin was used to mount the film and observe it under an optical microscope,^26^ and calculated the proportion of neurons of positive Nissl staining in the total number of neurons under the microscope field. Similarly, the count was done by an observer blinded to grouping.

### 2.11 Triphenyl tetrazolium chloride staining

The rats were sacrificed 24 h after MCAO/R. The brain was taken out and pre‐cooled at a low temperature (−80°C) for 8 min. Starting from the frontal pole, the brain was coronally sliced into five 2‐mm‐thick pieces. Then, the sections were immersed in a 2.0% 2,3,5‐triphenyltetrazolium chloride solution at 37°C for 30 min and then washed three times with PBS. The sections were fixed in 10% formalin and photographed with a digital camera. Infarct volumes were measured by a blinded observer using Image J software on TTC stained sections. Infarct volumes were corrected for brain edema by reporting the volume of the contralateral hemisphere minus the non‐infarcted volume of the ipsilateral hemisphere.

### 2.12 Neurological scoring

At 24 h after MCAO/R, neurological tests were conducted on 12 rats per group to assess behavioral impairments. Behavioral performance was scored according to a previously published scoring system,^14^ which was a revised version of the Bederson score. The recording of the data was completed by two blind researchers.

### 2.13 Rotarod test

Before the formal test, each rat was trained three times a day for 3 consecutive days. The motor function of rats was evaluated in 1, 3, 5, 7, and 14 of MCAO/R. The rotating rod with a diameter of 10 cm was increased from 4 rpm to 30 rpm within 60s and maintained for 300 s at the longest. When the rats lost balance and fell off the start sensor, the stopping time of the machine was recorded. The evaluation of three rats could be completed at the same time, and there was a partition between them so it would not affect each other. The recording of the data was completed by two blind researchers.

### 2.14 Adhesive removal test

After training, the rats that could remove the stickers within 10 s were selected and included in the experimental group. First, let the experimental rat get acquainted with the test environment, then stick two pieces of adhesive paper dots on the bilateral forelimbs of the rats and place them gently in the test cage. The contact was recorded and removal of each limb was done five times a day for 3 consecutive days. Rats in all experimental groups were tested on all test days after MCAO/R.^27^ The recording of the data was completed by two blind researchers.

### 2.15 Primary neuron‐enriched cultures

Using poly‐D‐lysine (Sigma, USA) pre‐coated Petri dishes and well plates, the embryonic brains of 17‐day‐old rats were aseptically extracted from pregnant rats, and the brains were separated under the microscope on ice. The meninges and blood vessels were then rinsed with PBS, and then the harvested brain tissue was digested with 0.25% trypsin (Thermo Fisher, USA) at 37°C for 6 min. Then, the digested brain tissue was washed with phosphate‐buffered saline (PBS), and then the resulting brain suspension was set at a speed of 1500 rpm. After centrifugation for 5 min, the supernatant was discarded, and the resuspended cells were seeded into Petri dishes, 6‐well plates, and 12‐well plates. Subsequently, the isolated cortical neurons were cultured in a neurobasal medium (Gibco) with 0.5 mM GlutaMAX (Gibco), 2% B‐27(Gibco), 50 U/ml streptomycin, and 50 U/ml penicillin (Invitrogen). Finally, the neurons were placed in a constant temperature of 37°C incubators with humidified air containing 5% CO_2_. We changed the medium every 2 days for 1 week, after which neurons were harvested for subsequent analysis.^28^

### 2.16 Establishing an oxygen–glucose deprivation/reoxygenation (OGD/R) model in vitro

Replace the neurobasal medium with sugar‐free DMEM (Gibco) and transfer the cells to an atmospheric incubator containing 5% CO_2_ and 95% N_2_ and incubate at 37°C for 2 h. After that the neurons were relieved of hypoxia and sugar‐free state, replaced with a neurobasal medium, and placed in a 5% CO_2_ atmosphere incubator.^28^

### 2.17 Mitochondrial membrane potential detection

We used 0.5 ml JC‐1 staining working solution (Beyotime, Shanghai, China) per 500,000–1,000,000 cells. Added 8 ml ultra‐pure water to each 50 μl JC‐1 (200×) to dilute JC‐1, then added 2 ml JC‐1 staining buffer (5×), and mixed well to become the JC‐1 staining working solution. According to the ratio of adding 4 ml of distilled water to each 1 ml of JC‐1 staining buffer (5×), an appropriate amount of JC‐1 staining buffer (1×) was prepared and placed in an ice bath. Dilute the CCCP (10 mM) provided in the kit at a ratio of 1:1000 and add it to the cell culture medium to treat the cells for 20 min, which was the negative control group. Aspirated the culture medium in the six‐well plate, washed it once with PBS, and then added 1 ml of cell culture medium. One ml of JC‐1 dyeing working solution was added, mixed well, and incubated for 20 min at 37°C in a cell incubator. Then, we aspirated the supernatant, washed twice with JC‐1 staining buffer, and observed under a fluorescence microscope.^29^

### 2.18 Fluo‐4 AM staining

First, take an appropriate amount of Fluo‐4 AM (Beyotime) store and dilute it to the 0.5–5 μM working solution with PBS. The culture neurons were removed from the culture medium and washed three times with PBS. One ml of Fluo‐4 AM working solution was added to cover the bottom of the 12‐well plate, which was incubated at 37°C for 60 min to load the fluorescent probe. Then, wash three times with PBS and incubate for 20–30 min after washing to ensure that Fluo‐4 AM is wholly transformed into Fluo‐4 in the cells. A laser microscope was used to determine the changes in the intracellular calcium ion concentration.

### 2.19 Live/dead cellular staining in vitro

Live/dead cell staining was used to observe cell apoptosis. Using calcein‐AM/propidium iodide (PI) double‐stain kit (Thermo Fisher Scientific) to detect in vitro, the cultured neurons were washed to remove active esterase in the culture. The prepared working reagent containing calcein‐AM and PI to the cells was added and incubated for 30 min. The apoptotic ratio was calculated under fluorescence microscopy (Nikon).^25^

### 2.20 Co‐immunoprecipitation analysis

The sample of the brain was prepared as described for the western blotting. First, the brain samples were lysed in cell lysis buffer for western blotting and IP (Beyotime). The above process was completed on the magnetic stand. Second, each mixture was incubated with Sam50 antibody or normal rabbit IgG (Cell Signal Technology). Protein A/G immunoprecipitation magnetic beads, which were well washed, were then added to each group of the protein mixture, and the protein––antibody bead mixture was incubated for 8 h at 4°C with rotary agitation. Third, the mixtures were washed five times with cell lysis buffer and then denatured with 30 μl 2 × SDS loading buffer. Finally, western blotting was performed to detect the relative protein levels of Mic19 and Sam50.^30^

### 2.21 Statistical analysis

Graph Pad Prism 8.0 (Graph Pad, United States) was used for statistical analyses, and all the results were expressed as means ± standard deviation (mean ± SD). All data were tested for normal distribution through the Anderson–Darling test, D'Agostino–Pearson test, Shapiro–Wilk test, and Kolmogorov–Smirnov test. Only data with a normal distribution could be counted. Ordinary one‐way ANOVA and two‐way ANOVA were used to analyze differences between multiple groups, while the Tukey test was used to analyze differences between two groups. Values of *p* < 0.05 were considered statistically significant.

## 3 RESULTS

### 3.1 General observations in rats

**FIGURE 1:**
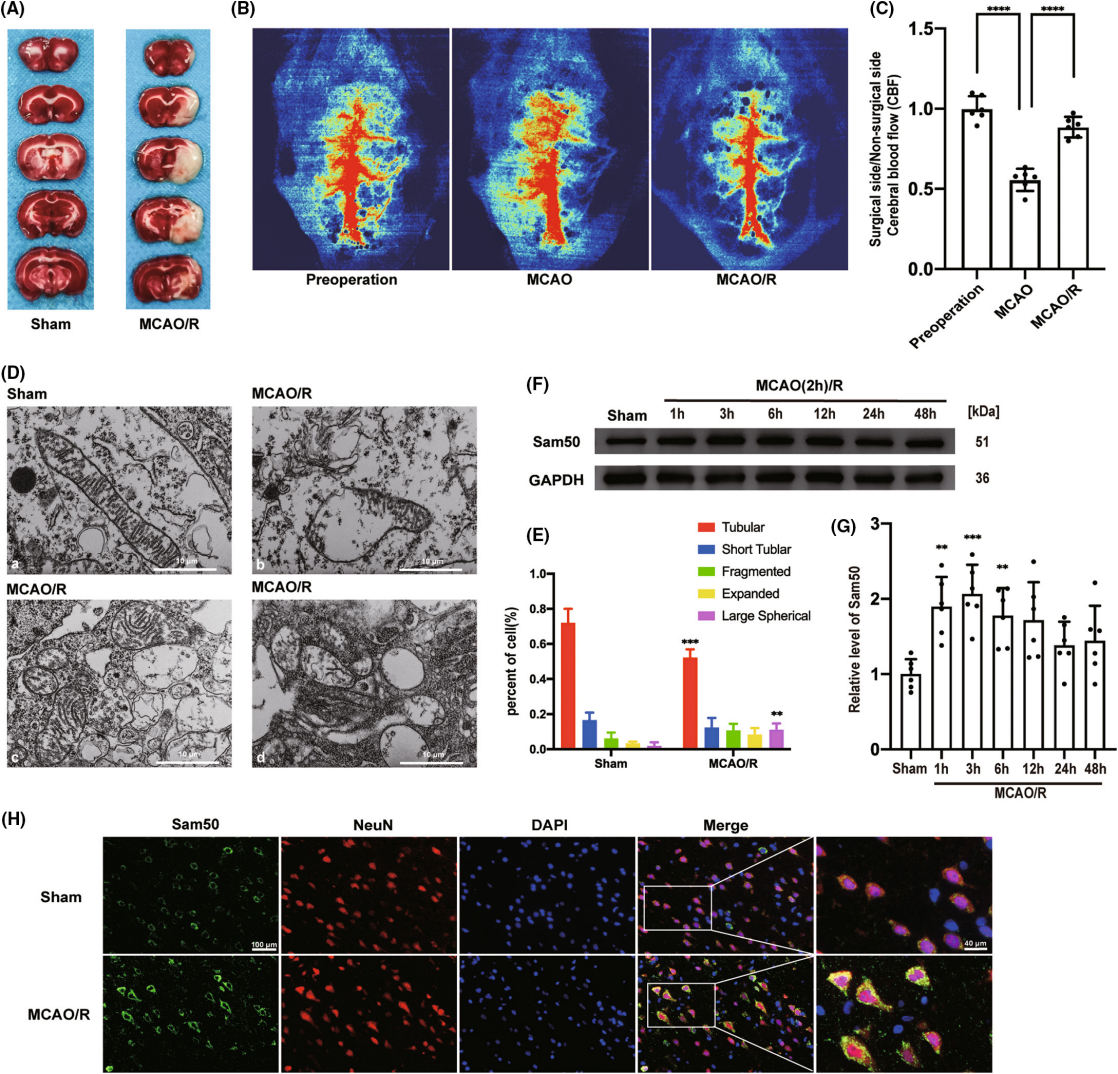
Mitochondrial damage and Sam50 protein levels increased in penumbra neurons after MCAO/R in rats. (A) Representative coronal brain sections at the scheduled times after MCAO/R. (B) Speckle imaging of cerebral blood flow before the operation, after MCAO, and after MCAO/R. (C) Statistical results of cerebral blood flow speckle imaging of preparation, after MCAO and after MCAO/R (****p < 0.0001 MCAO vs. pre‐operation group, ****p < 0.0001 MCAO/R vs. MCAO, *n* = 6). (D) Electron microscopy of mitochondria before and after MCAO/R, (a) normal structure of mitochondria in the sham group, (b) collapse of the crista junctions (CJs) found in the MCAO/R group, (c) star‐shaped crista of the mitochondria of MCAO/R group, (d) and complete collapse of the inner mitochondrial membrane of MCAO/R group. (E) Percentage statistics of different structures of crista in cells (tubular crista: ***p < 0.001: *p* = 0.0004, MCAO/R vs. sham; large spherical crista: **p < 0.01, MCAO/R vs. sham: *p* = 0.0027; *n* = 6.). (F, G) Western blot analysis and quantification of Sam50 in penumbra tissue after MCAO/R (**p < 0.01, 1 h: *p* = 0.0017, ***p < 0.001, 3 h: *p* = 0.0002, **p < 0.01, 6 h: *p* = 0.0074 vs. sham group, *n* = 6). (H) Double immunofluorescence analysis was performed with antibodies against Sam50 (green) and a neuronal marker (NeuN, red) in brain sections. Nuclei were fluorescently labeled with 4,6‐diamino‐2‐phenylindole DAPI (blue). Scale bar =100 μm. All data were displayed as means ± SD, *n* = 6, and differences were calculated with ordinary one‐way ANOVA.

There was no significant difference in weight, blood pressure, and body temperature in the experimental group compared with the sham group. In the sham group, the mortality rate was 0% (0/42) in rats, while in the experimental group, the mortality rate was 13.0% (29/221). For the specific mortality of each group of animals, please refer to Table S2. We showed the results of representative TTC staining in the sham and MCAO/R groups (Figure 1A). The CBF showed a decrease in cerebral blood flow after the right middle cerebral artery occlusion of rats and the recovery of middle cerebral blood flow after reperfusion (Figure 1B). We calculated the ratio of local blood flow between the surgical side and the non‐operating side and showed the quantitative display of cerebral blood flow changes after MCAO and MCAO/R (Figure 1C).

### 3.2 Mitochondrial damage and Sam50 protein levels increased in penumbra neurons after MCAO/R in rats

In the beginning, we found significant damage to the mitochondrial structure in rats' neurons after MCAO/R (Figure 1D). The mitochondrial morphology of the sham group was normal (Figure 1D). In the mitochondrial morphology after MCAO/R, we could see that the mitochondrial membrane of the MCAO/R group collapsed and irregular forms of the mitochondrial crista (Figure 1D). We counted different types of mitochondrial crista, including tubular crista, short tubular crista, fragmented crista, expanded crista, and large spherical crista. We found a marked decrease in the number of tubular cristae and an increase in the number of large spherical crista (Figure 1E).

So, we used western blotting to investigate changes in Sam50 protein levels within brain tissues after MCAO/R induction in rats. We found that the Sam50 protein significantly increased after MCAO/R treatment in rats and peaked at 3 h (Figure 1F). Then, we showed a statistical chart of the relative level of Sam50 of western blotting (Figure 1G). In order to better demonstrate the change in the expression content of Sam50, we performed immunofluorescent staining with NeuN (a neuron‐specific marker) and DAPI (a nucleus marker) after MCAO/R 3 h. The fluorescence intensity of Sam50 in the MCAO/R group was higher than that in the sham group (Figure 1H).

### 3.3 Sam50 mitigated MCAO/R‐induced neuronal apoptosis and neurodegeneration in rats

**FIGURE 2:**
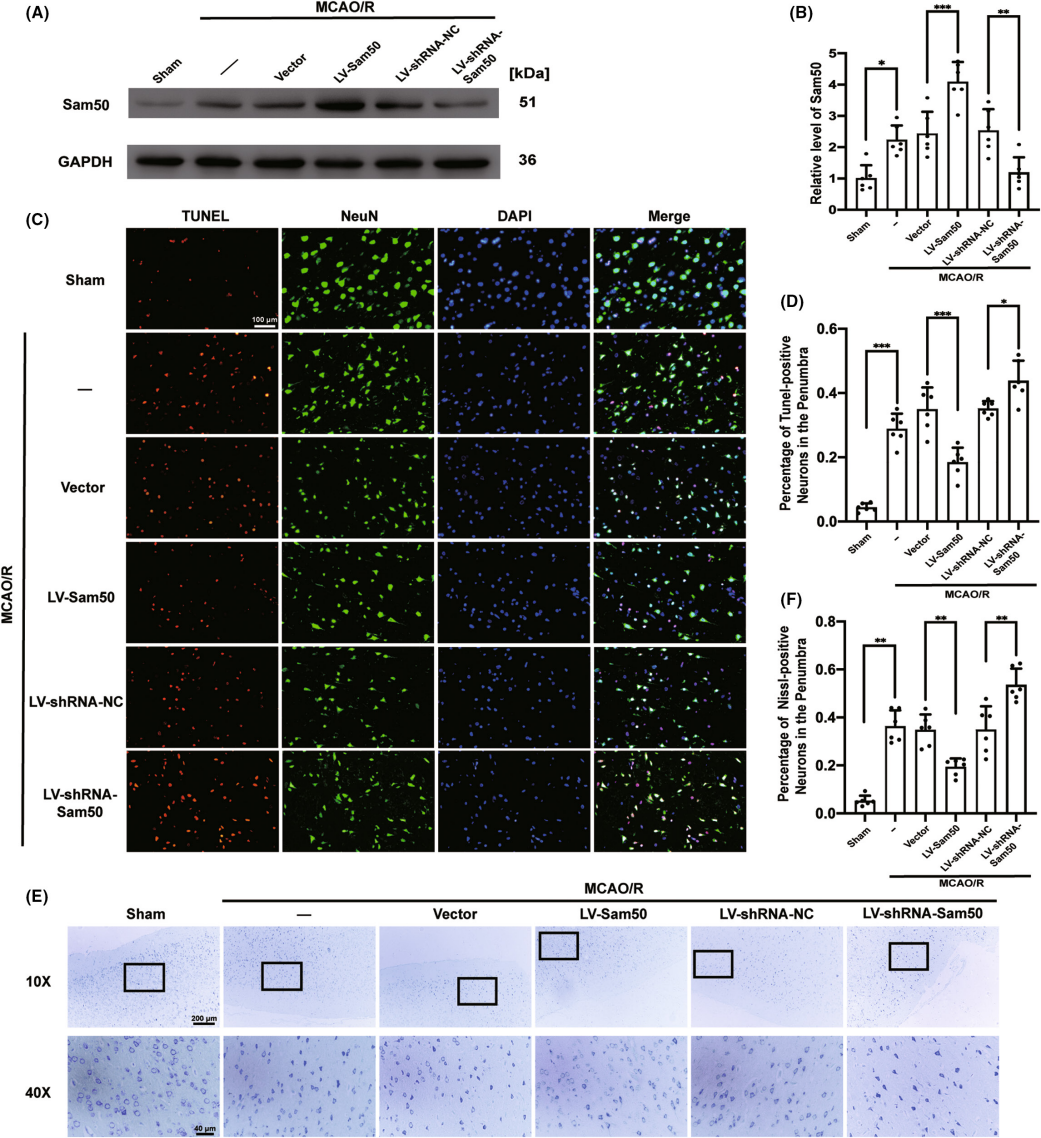
Sam50 mitigated MCAO/R‐induced neuronal apoptosis and neurodegeneration in rats. (A, B) Western blot analysis and quantification of the effects of overexpression and knockdown of Sam50 (*p < 0.05 vs. sham: *p* = 0.0109; ***p < 0.001, LV‐Sam50 vs. Vector: *p* = 0.0004; **p < 0.01, LV‐shRNA‐NC vs. LV‐shRNA‐Sam50: *p* = 0.0048; *n* = 6). (C, D) Double staining for terminal deoxynucleotidyl transferase‐mediated dUTP nick end labeling (TUNEL) (red) neuronal marker (NeuN, green), nuclei were fluorescently labeled DAPI (blue), and scale bar =100 μm (***p < 0.001 vs. sham: *p* = 0.0007; ***p < 0.001: *p* = 0.0005, LV‐Sam50 vs. Vector; *p < 0.05, LV‐shRNA‐NC vs. LV‐shRNA‐Sam50: *p* = 0.0385, *n* = 6). (E, F) Nissl staining and statistics (select the black rectangle area to magnify) and statistic of Nissl staining positive ratio (**p < 0.01, vs. sham: *p* = 0.0073; **p < 0.01, LV‐Sam50 vs. Vector: *p* = 0.0028; **p < 0.01, LV‐shRNA‐NC vs. LV‐shRNA‐Sam50: *p* = 0.0086; *n* = 6). All data were displayed as means ± SD; *n* = 6, and differences were calculated with ordinary one‐way ANOVA.

In order to further investigate the role of Sam50 in the MCAO/R model of rats, we use LV to overexpress and lower Sam50 in brain neurons in rats. The effect of LV‐Sam50 and LV‐shRNA‐Sam50 was confirmed by western blotting (Figure 2A). Relevant statistical charts were displayed (Figure 2B). Next, we assessed the damage to neurons under intervention conditions using Nissl staining and TUNEL staining. The results of TUNEL staining showed that after MCAO/R treatment, the proportion of neuronal apoptosis in the penumbra of rats was significantly increased (Figure 2C). This performance was reversed after the overexpression of Sam50, and the proportion of TUNEL positive decreased. Moreover, after using LV to reduce the expression of Sam50, the TUNEL‐positive rate increased significantly (Figure 2D). As for Nissl staining, we are concerned about the positive proportion of neurons in the penumbra zone under the white light microscopes. Our result was that the MCAO/R group had a higher proportion of neurons that tested positive for Nissl staining than the sham group. In the overexpression group, the proportion of neurons positive for Nissl staining decreased. On the contrary, the proportion of positive neurons that for Nissl staining increased after Sam50 was knockdown (Figure 2E,F).

### 3.4 Sam50 rescued MCAO/R‐induced infarction area and neurobehavioral in rats

**FIGURE 3:**
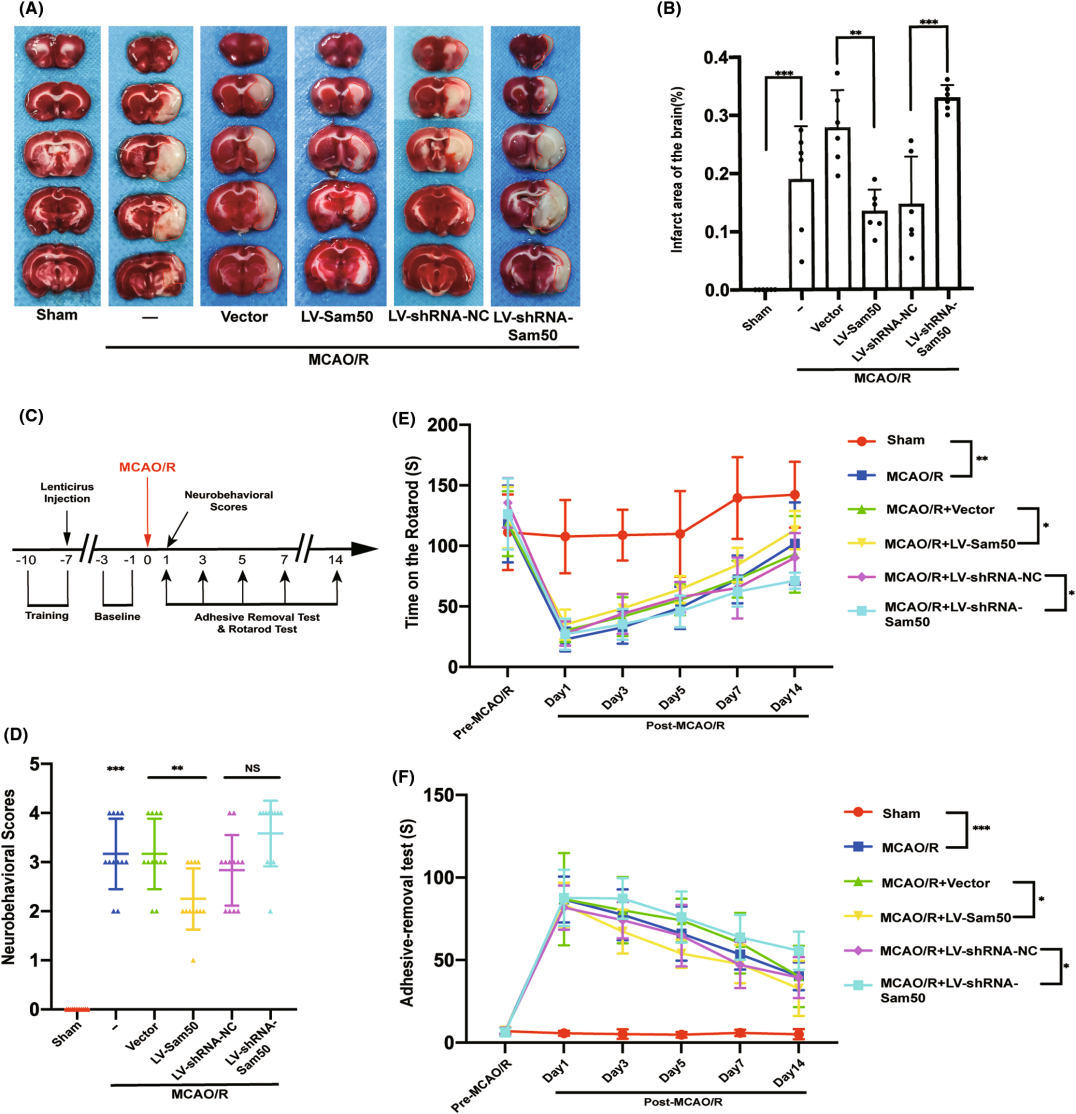
Sam50 rescued MCAO/R‐induced infarction area and neurobehavioral in rats. (A, B) Triphenyl tetrazolium chloride (TTC) staining and statistics of the effects of overexpression and knockdown of Sam50 (***p < 0.001, vs. sham: *p* = 0.0004; **p < 0.01: *p* = 0.0027, LV‐Sam50 vs. Vector; ***p < 0.001, LV‐shRNA‐NC vs. LV‐shRNA‐Sam50: *p* = 0.0001, *n* = 6). (C) Behavioral experiment‐related process. (D) Neurological Scoring (***p < 0.001, vs. sham: *p* = 0.0003; **p < 0.01, LV‐Sam50 vs. Vector: *p* = 0.0085; *p* > 0.05, LV‐shRNA‐NC vs. LV‐shRNA‐Sam50, *n* = 12). (E) Rotarod test at pre‐MCAO/R, 1, 3, 5, 7, and 14 days of post‐MCAO/R (**p < 0.01, vs. sham: *p* = 0.0088; *p < 0.05, LV‐Sam50 vs. Vector: *p* = 0.0256; *p < 0.05, LV‐shRNA‐NC vs. LV‐shRNA‐Sam50: *p* = 0.0462, *n* = 12). (F) Adhesive removal time (***p < 0.001, vs. sham: *p* = 0.0007; *p < 0.05, LV‐Sam50 vs. Vector: *p* = 0.0124; *p < 0.05, LV‐shRNA‐NC vs. LV‐shRNA‐Sam50: *p* = 0.0256; *n* = 12). All data were displayed as means ± SD, *n* = 6 for (A, B), *n* = 12 for (C, D, E, F), and differences were calculated with two‐way ANOVA for (E, F) and ordinary one‐way ANOVA for (B, D).

In order to verify the difference between infarction areas under different intervention conditions, we chose TTC staining. We can conclude that after the overexpression of Sam50, TTC staining showed a significant decrease in infarction area compared to the vector group. After sam50 was knockdown, the area of core infarction after MCAO/R in rats' brains increased significantly compared to the control group (Figure 3A,B). Next, we investigated the relationship between rats' behavior and Sam50 protein levels. The flow of behavioral experiments was reflected (Figure 3C). Compared with the sham group, the neurobehavioral scores were increased after MCAO/R induction, and that was more exacerbated with the Sam50 knockdown. On the contrary, overexpression of Sam50 significantly reduced MCAO/R‐induced neurological damage (Figure 3D). In addition, we used the Rotarod and the adhesive removal test to detect the balance and sensory function changes in rats in the intervention state. The time spent on the Rotarod in the MCAO/R group was significantly reduced, and the rats in the Sam50‐OE group were able to persist longer than the vector group. When the Sam50 was knockdown, the rats stayed on the Rotarod machine for less time than the LV‐shRNA‐NC group (Figure 3E). As for the adhesive removal test, the rats feeling after MCAO/R were significantly reduced compared to the sham group. Overexpression of Sam50 could alleviate somatosensory dysfunction induced by MCAO/R in rats. On the contrary, after reducing the expression of Sam50, the sensory impairment of rats increased (Figure 3F).

### 3.5 Sam50 could improve MCAO/R‐induced mitochondrial structure destruction in rats

**FIGURE 4:**
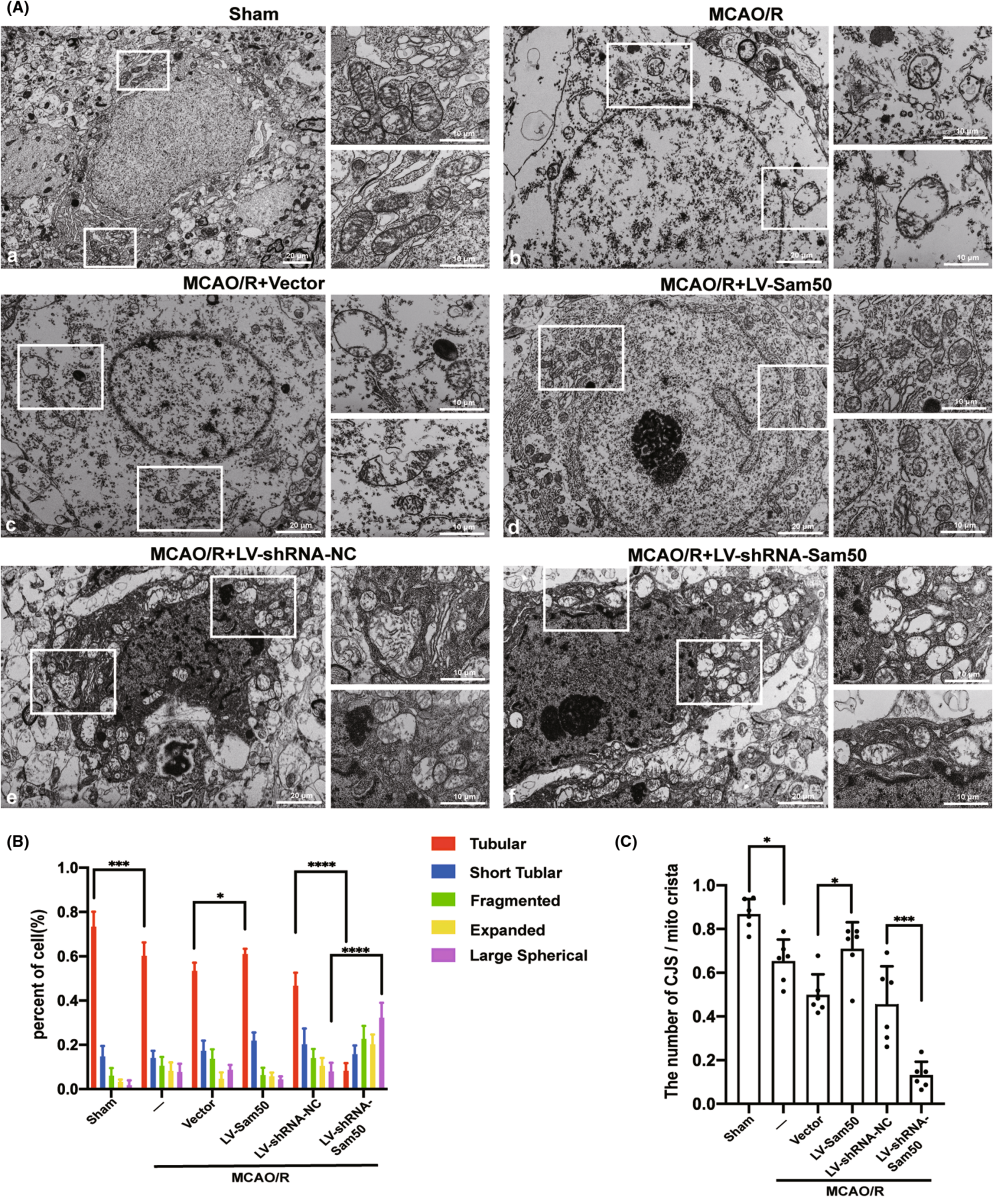
Sam50 could improve MCAO/R‐induced mitochondrial structure destruction in rats. (A) Mitochondrial structure under different conditions, (a) relatively intact mitochondrial membrane structure in the sham group, (b, c, e) inner membrane collapse, CJs loss and reduction in the MCAO/R group, MCAO/R + Vector group, and MCAO/R + LV‐shRNA‐NC group, (d) mitochondrial structural damage reduced in the MCAO/R + LV‐Sam50 group, and (f) increased mitochondrial damage in the MCAO/R + LV‐ shRNA‐Sam50 group. (B) Percentage statistics of different structures of crista in cells (tubular crista: ***p < 0.001, vs. sham: *p* = 0.0009; *p < 0.05, LV‐Sam50 vs. Vector: *p* = 0.0215; ****p < 0.0001, LV‐shRNA‐NC vs. LV‐shRNA‐Sam50, large spherical crista: ****p < 0.0001, and LV‐shRNA‐NC vs. LV‐shRNA‐Sam50, *n* = 6). (C) Statistics of the number of CJs/ mitochondrial crista (*p < 0.05, vs. sham: *p* = 0.0225; *p < 0.05, LV‐Sam50 vs. Vector: *p* = 0.0269; ***p < 0.001, and LV‐shRNA‐NC vs. LV‐shRNA‐Sam50: *p* = 0.0002, *n* = 6). All data were displayed as means ± SD, *n* = 6, and differences were calculated with two‐way ANOVA for (A, B) and ordinary one‐way ANOVA for (C).

In order to explore the structural changes in mitochondria under different intervention conditions, we used electron microscopy to observe structural changes in mitochondria between different groups. Compared with the sham group, the mitochondrial structure in the MCAO/R group was obviously abnormal. We could see mitochondrial membrane and crista collapse. Compared with the group vector and MCAO/R, the vector group of mitochondria also experienced damage to the membrane structure of mitochondria. After overexpression of the Sam50 protein, the structure of mitochondria in OE groups improved significantly compared to that of vector groups. When the Sam50 protein content was knockdown using LV, mitochondrial structural damage was significantly increased, and we could see a large number of mitochondrial membranes and crista falling off and disintegrating (Figure 4A). The different types of mitochondrial crista were counted separately under different intervention conditions (Figure 4B), including tubular crista, short tubular crista, fragmented crista, expanded crista, and large spherical crista. Compared with group sham and group MCAO/R. The number of tubular cristae was significantly reduced, the number of tubular cristae in the LV‐Sam50 group was significantly higher than in the vector group, and group LV‐shRNA‐Sam50 had a much smaller number of tubular crista than group LV‐shRNA‐NC. We also compared the ratio of the number of CJs to the number of cristae between different groups, the number of CJs decreased after ischemic reperfusion, and the number of CJs plummeted again after the Sam50 knockdown. This phenomenon improved after the Sam50 was raised (Figure 4C).

### 3.6 OGD/R increased Sam50 protein levels in neurons and Sam50 rescue OGD/R‐induced neuronal cell death

**FIGURE 5:**
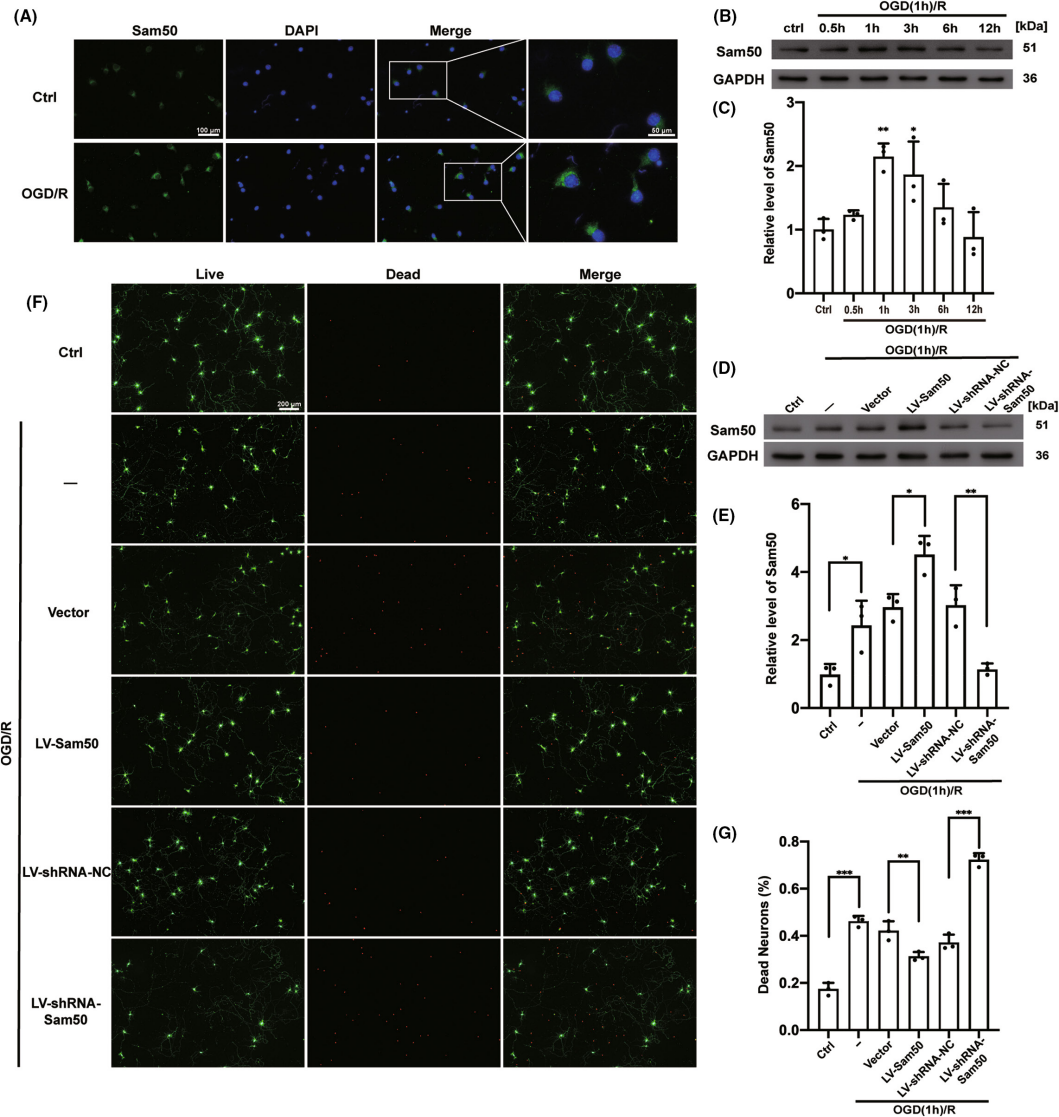
OGD/R increased Sam50 protein levels in neurons and Sam50 rescued OGD/R‐induced neuronal cell death. (A) Immunofluorescence analysis was performed with antibodies against Sam50 (green), and nuclei were fluorescently labeled with 4,6‐diamino‐2‐phenylindole DAPI (blue). Scale bar =100 μm, *n* = 3. (B, C) Western blot analysis and quantification of Sam50 of neurons after OGD/R (**p < 0.01, 0.5 h: *p* = 0.0044, *p < 0.05 vs. Ctrl group: P = 0.0283. *n* = 3). (D, E) Western blot analysis and quantification of the effects of overexpression and knockdown of Sam50 in vitro (*p < 0.05 vs. Ctrl: *p* = 0.0296; *p < 0.05, LV‐Sam50 vs. Vector: *p* = 0.0183; **p < 0.01, LV‐shRNA‐NC vs. LV‐shRNA‐Sam50: *p* = 0.0045; *n* = 3). (F, G) Live/dead cell staining: calcein‐AM (green means cells are alive) and propidium iodide (PI) (red means cells are death) (***p < 0.001 vs. Ctrl: *p* = 0.0004; **p < 0.01, LV‐Sam50 vs. Vector: *p* = 0.0069; ***p < 0.001, LV‐shRNA‐NC vs. LV‐shRNA‐Sam50: *p* = 0.0003; scale bar =200 μm, *n* = 3). All data were displayed as means ± SD of three independent experiments performed in triplicate, differences were calculated with ordinary one‐way ANOVA.

To further prove the role of Sam50 in vitro models, we built an OGD/R model of primary cultured neurons. We found an increased expression of Sam50 with OGD/R intervention in immunofluorescence (Figure 5A). This was also confirmed in the western blot, and we found the peaks appeared earlier in 1 h (Figure 5B,C). Next, we intervened in the expression content of Sam50. We used lentiviruses to overexpress and knockdown Sam50 (Figure 5D,E). To investigate the effect of Sam50 expression on neuronal death, we used live–dead cell staining. The results showed that more dead neurons (red) were detected in the MCAO/R group compared with those in the control group, and the number of dead cells was exacerbated after Sam50 knockdown and reversed after Sam50 overexpression (Figure 5F,G).

### 3.7 Sam50 improved OGD/R‐induced mitochondrial membrane potential (MMP) decrease and Ca^2+^ overload in neurons

**FIGURE 6:**
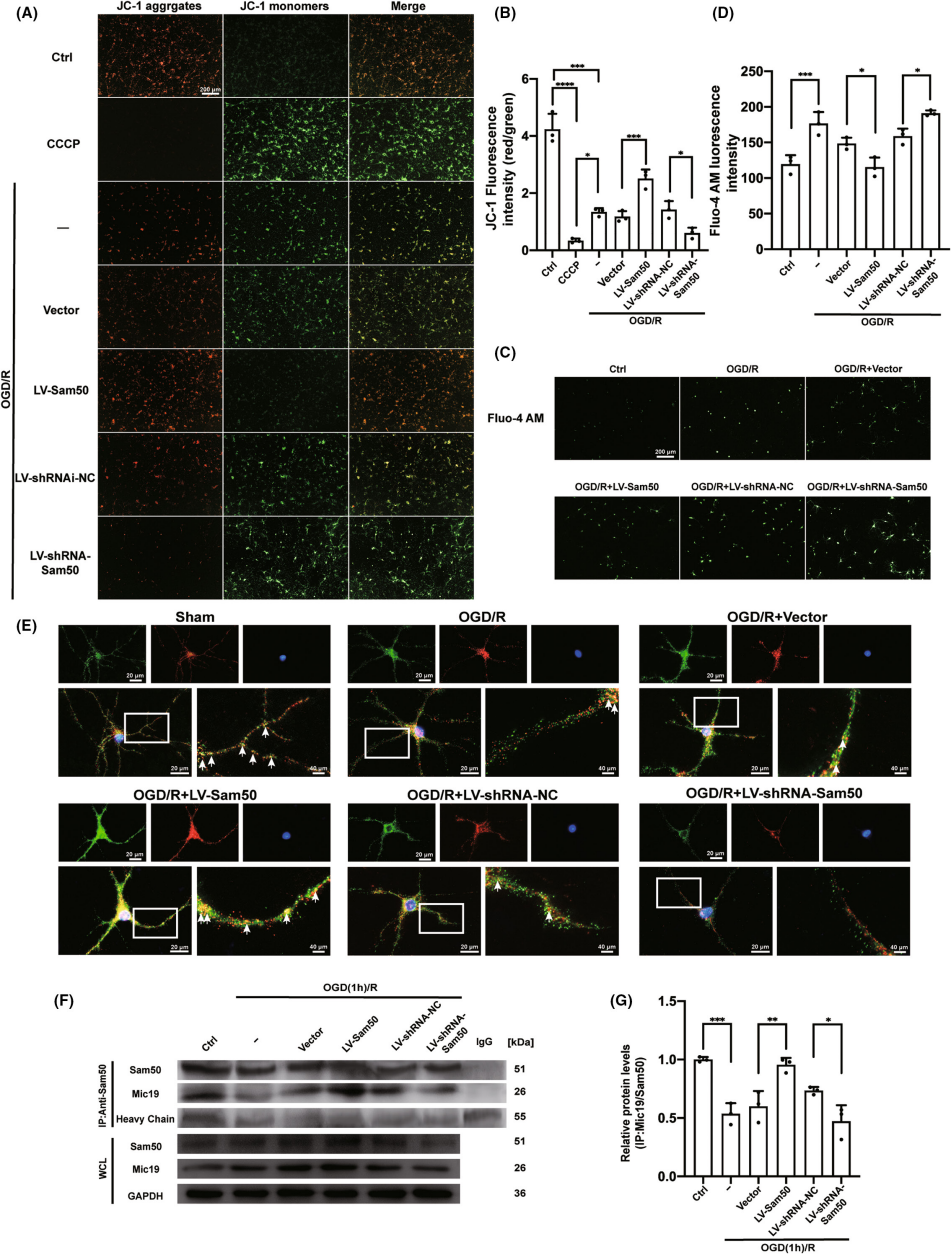
Changes in mitochondrial membrane potential (MMP) and Ca^2+^ content under different expressions of Sam50 after OGD/R. (A, B) JC‐1 staining: red (JC‐1 aggregates) represents higher MMP, green (JC‐1 monomers) represents lower MMP (***p < 0.001, OGD/R vs. Ctrl: *p* = 0.0007; ****p < 0.0001, CCCP vs. Ctrl; *p < 0.05, CCCP vs. OGD/R: *p* = 0.0113; ***p < 0.001, LV‐ Sam50 vs. Vector: *p* = 0.0010; *p < 0.05, LV‐shRNA‐NC vs. LV‐shRNA‐Sam50: *p* = 0.0471; scale bar =200 μm, *n* = 3). (C, D) Fluo‐4 AM staining: the fluorescence intensity is proportional to the accumulation of Ca2+ (***p < 0.001 vs. Ctrl: *p* = 0.0007; *p < 0.05, LV‐Sam50 vs. Vector: *p* = 0.0326; *p < 0.05, LV‐shRNA‐NC vs. LV‐shRNA‐Sam50: *p* = 0.0462; scale bar =200 μm, *n* = 3). (E) Double staining for Sam50 (green) and Mic 19 (red), and nuclei were fluorescently labeled DAPI (blue). The fluorescence intensity of the three co‐staining represents the connection strength of the Sam50–Mic19 axis. (F) Mic19/Sam50 interactions in neurons after OGD/R. (G) Quantitative analysis was performed. The black dots represent individual data in each group. All data were displayed as Means ± SD; mean values for Ctrl group were normalized to 1.0; (***p < 0.001 vs. Ctrl: *p* = 0.0004; **p < 0.01, LV‐Sam50 vs. Vector: P = 0.0045; *p < 0.05, LV‐shRNA‐NC vs. LV‐shRNA‐Sam50: *p* = 0.0372, *n* = 3).

To further explore the effects of Sam50 on mitochondrial function, JC‐1 staining was used to detect the mitochondrial membrane potential (MMP). Usually, in normal mitochondria, JC‐1 aggregates with red fluorescence, whereas this signal was converted into green fluorescence in damaged mitochondria. Under the intervention conditions, the red‐fluorescence intensity of the OGD/R group was decreased, and the green‐fluorescence intensity increased. Which indicated a reduction in the MMP and an opening in the mitochondrial permeability transition pore (MPTP). Compared with the corresponding virus overload group, in the OGD/R+ LV‐Sam50 group, this effect was reversed; in contrast, this effect was exacerbated in the OGD/R+ LV‐shRNA‐Sam50 group (Figure 6A,B). Next, in order to investigate the accumulation of Ca^2+^ in cells, we used Fluo‐4 AM to stain neuronal cells. The higher the fluorescence intensity, the higher the Ca^2+^ content. After OGD/R, there was a significant increase in Ca^2+^ in neurons. The fluorescence intensity of Fluo‐4 AM decreased after Sam50 was expressed, while the fluorescence intensity decreased after Sam50 was knockdown (Figure 6C,D).

### 3.8 Sam50–Mic19 axis was interrupted by I/R injury

Finally, we wanted to explore the connection between Sam50 and Mic19. We dyed Sam50, Mic19, and DAPI together. After OGD/R, the fluorescence intensity of Sam50 increased. However, the fluorescence intensity of the Merge images of Sam50, Mic19, and DAPI decreased. After the Sam50 overexpression was treated, the fluorescence intensity of co‐dye increased significantly. Instead, the fluorescence intensity of the Merge image decreased after the Sam50 was lowered. This proves that expressing the Sam50 increases the connection between the Sam50 and Mic19 (Figure 6E). We used co‐immunoprecipitation experiments to confirm the change in the intensity of the effect between Mic19 and Sam50 again. The obtained results corroborated the results of immunofluorescence. The interaction strength of Mic19 and Sam50 decreases in neurons after oxygen–glucose deprivation. Overexpression of Sam50 improves the interaction strength between Mic19 and Sam50. On the contrary, the effect was further reduced (Figure 6F,G).

## 4 DISCUSSION

Cerebral ischemia (CI) is one of the strokes which can lead to disability and even death. The CI is mainly caused by acute brain vascular occlusion. Mitochondrial damage has been reported to be an essential factor for induced cerebral ischemia. In this study, our results demonstrated that cerebral ischemia led to abnormal mitochondrial structure, such as the collapse of the inner mitochondrial membrane and the abnormal shape of the crista junction (CJ).

The mitochondrial sorting and assembly machinery (SAM; also called the “topogenesis of mitochondrial outer membrane β‐barrel,” or TOB complex).^31^, ^32^, ^33^ is assembled by three components: the core subunit Sam50 (Tob55) and two peripheral membrane proteins, Sam35 and Sam37 (Metaxin1 (MTX1) and Metaxin2 (MTX2) in mammals).^34^ Sam50 acts as a barrel protein for the outer membrane of the mitochondria, which has a variety of functions such as stabilizing mitochondrial structure and assembling mitochondrial outer membrane barrel protein.^12^, ^35^, ^36^, ^37^ Sam50 depletion, but not Metaxin2 depletion, could trigger abnormal mitochondrial morphology and cristae structures.^38^, ^39^, ^40^ In this experiment, we validated the elevated expression of Sam50 in ischemia–reperfusion models in vivo and in vitro. We think that following an ischemic stroke event, with mitochondrial damage and disruption of the connection between Sam50 and Mic19, neuronal mitochondria may exhibit a stress response that results in a short‐term elevation of Sam50. However, with the gradual consumption and the lack of timely supplementation after injury, the protein level of Sam50 gradually decreased. More Sam50s are required to re‐establish the connection between Sam50 and Mic19. Furthermore, we found that overexpression of Sam50 reduces ischemia–reperfusion damage, such as reduced neuronal apoptosis and degeneration, infarct volume, and mitochondrial structural damage under electron microscopy, and improved the behavior of rats. In vitro, we also observed a similar phenomenon. After overexpression of Sam50, neuronal mortality decreased and mitochondrial membrane potential increased.

In human mitochondria, the inner membrane MICOS complex interacts with the outer membrane sorting and assembly machinery (SAM) complex to form the mitochondrial intermembrane space bridging complex (MIB, supercomplex for mediating mitochondrial outer‐ and inner‐membrane contact).^41^ According to research reports, Mic19 directly interacts with mitochondrial outer membrane protein Sam50 and inner membrane protein Mic60 to form the Sam50–Mic19–Mic60 axis, which dominantly connects SAM and MICOS complexes to assemble MIB.^12^ Mic19 cleavage disrupts the Sam50–Mic19–Mic60 axis and disassembles MIB supercomplex.^12^ We demonstrated the protective effect of Sam50 on mitochondria during I/R injury. After I/R, the Sam50–Mic19–Mic60 axis fractured, and this process could be reversed after Sam50 overexpression. Therefore, Sam50 improved nerve injury after I/R, and its possible mechanisms may be closely related to maintaining the stability of the Sam50–Mic19 axis.

This experiment also has some shortcomings. First, this experiment uses the rat MCAO/R model to mimic the occurrence of ischemic stroke, but normal stroke can also occur in blood vessels other than the middle cerebral arteries, and the blood vessels may not be re‐communicated. Second, due to the physiological cycle of female rats, only male rats were used in this study. However, sex differences have been reported in cerebral metabolism, cerebrovascular and blood flow, response to stroke, and stroke outcomes.^42^, ^43^, ^44^, ^45^, ^46^ Then, we use the OGD/R model in vitro, which ignores other influencing factors of neurons in the brain such as heme, plasmin, and inflammatory factors; whether these components also induce mitochondrial dysfunction remains unclear. Finally, we only focus on the mitochondrial structure of effects of Sam50 in ischemic stroke conditions. Sam50 is the core component of the SAM complex, and the SAM complex is responsible for inserting the barrel into the mitochondrial outer membrane.^35^ In contrast to bacteria, mitochondria are predicted to make only four types of β‐barrel outer membrane protein: Sam50, Tom40, VDAC, and Mdm10 (Mdm10 is not expressed in mammals).^37^ The above several barrel proteins have their functions. Whether the expression of Sam50 further affects the function of the above proteins needs to be further explored.

## 5 CONCLUSION

Sam50 was increased after I/R injury and showed neuroprotective effects in I/R‐induced brain injury by maintaining mitochondrial structure, which may be a new target for the treatment of ischemic stroke.

## AUTHOR CONTRIBUTIONS

XL and HW participated in the design of this study. XY and JW performed the experiments and wrote the study. SY and HS helped to design the analysis strategy and implement the analysis and constructive discussion. HL and GC helped to conduct the literature review. XL and HW reviewed and edited the manuscript. All authors have critically revised the manuscript and approved the final version.

## FUNDING INFORMATION

This work was supported by the Natural Science Foundation of Jiangsu Province under Grant (No. BK20220096 and No. BK20211552) and Suzhou Science and Technology (No. SKJY2021058).

## CONFLICT OF INTEREST

The authors declare that they have no competing interests.

## Supporting information


Appendix S1

Appendix S2

## Data Availability

The datasets generated and/or analyzed during the current study are not publicly available due to the confidential policy of our hospital but are available from the corresponding author upon reasonable request.
