# Supplementary material for: Sam50 exerts neuroprotection by maintaining the mitochondrial structure during experimental cerebral ischemia/reperfusion injury in rats
Source: CNS Neurosci Ther. 2022 Sep 8;28(12):2230–44. doi: 10.1111/cns.13967 (PMC9627377; doi:10.1111/cns.13967)
Supplement: Supplementary file 1 — Appendix S1 [file CNS-28-2230-s002.docx]

**Appendix Table S1 Mortality of rat in each experiment**

| **Group** | **total** | **dead** | **survived** | **time** | **Survival rate** |
| --- | --- | --- | --- | --- | --- |
| **Experiment 1** |  |  |  |  |  |
| Sham | 12 | 0 | 12 | - | 100.0% |
| MCAO/R 1 h | 7 | 1 | 6 | During surgery | 85.7% |
| MCAO/R 3 h | 8 | 2 | 6 | During surgery | 75.0% |
| MCAO/R 6 h | 14 | 2 | 12 | 2 after surgery | 85.7% |
| MCAO/R 12 h | 7 | 1 | 6 | After reperfusion for 1 h | 85.7% |
| MCAO/R 24 h | 8 | 2 | 6 | After reperfusion for 3 h and 6 h | 75.0% |
| MCAO/R 48 h | 7 | 1 | 6 | After reperfusion for 24 h | 85.7% |
| **Experiment 2** |  |  |  |  |  |
| Sham | 30 | 0 | 30 | - | 100.0% |
| MCAO/R | 35 | 5 | 30 | 3 during surgery and 2 after reperfusion | 85.7% |
| MCAO/R +Vector | 35 | 5 | 30 | 3 during surgery, 1 after reperfusion and 1 after injecting the virus | 85.7% |
| MCAO/R + LV-Sam50 | 33 | 3 | 30 | 2 during surgery and 1 after injecting the virus | 90.9% |
| MCAO/R + LV-shRNA-NC | 32 | 2 | 30 | 2 during surgery | 93.8% |
| MCAO/R + LV-shRNA-Sam50 | 35 | 5 | 30 | 3 during surgery and 2 after injecting the virus | 85.7% |

**Appendix Table S2 Antibody Reporting**

| **Antibody** | **RRID** | **Full name** | **Specificity** | **Citation** | **Type** | **Host** |
| --- | --- | --- | --- | --- | --- | --- |
| Sam50 | AB_2646937 | SAMM50 Polyclonal Antibody | Human, Mouse, Rat | **PA5-56969** | Polyclonal Antibody | Rabbit |
| Sam50 |  | Sam50 Antibody (SQ-7) | mouse, rat and  human | **sc-100493** | Monoclonal Antibody | mouse |
| Mic19 |  | Anti-MIC19 antibody | Mouse, Rat, Human | ab224565 | Polyclonal Antibody | Rabbit |
| GAPDH | [AB_2839421](https://antibodyregistry.org/search.php?q=AB_2839421) | GAPDH Antibody | Human, Mouse, Rat, Pig,Bovine, Goat, Monkey, Chicken | AF7021 | Polyclonal Antibody | Rabbit |
| anti-NeuN antibody |  | Anti-NeuN antibody - Neuronal Marker | Mouse, Rat, Human | ab104225 | Polyclonal Antibody | Rabbit |
| anti-NeuN antibody[1B7] |  | Anti-NeuN antibody [1B7] - Neuronal Marker | Mouse, Rat, Human | ab104224 | Monoclonal Antibody | mouse |
| Anti-rabbit IgG |  | Anti-rabbit IgG, HRP-linked Antibody | Rabbit | 7074S | Monoclonal Antibody | goat |
| Anti-mouse IgG |  | Anti-mouse IgG, HRP-linked Antibody | Mouse | 7076S | Monoclonal Antibody | horse |
| anti-Rabbit IgG, Alexa Fluor 488 | AB_2535792 | Donkey anti-Rabbit IgG (H+L) Highly Cross-Adsorbed Secondary Antibody, Alexa Fluor 488 | Rabbit | **A-21206** | Polyclonal Antibody | Donkey |
| anti-Rabbit IgG, Alexa Fluor 555 | AB_162543 | Donkey anti-Rabbit IgG (H+L) Highly Cross-Adsorbed Secondary Antibody, Alexa Fluor 555 | Rabbit | **A-31572** | Polyclonal Antibody | Donkey |
| anti-Mouse IgG, Alexa Fluor 488 | AB_141607 | Donkey anti-Mouse IgG (H+L) Highly Cross-Adsorbed Secondary Antibody, Alexa Fluor 488 | Mouse | **A-21202** | Polyclonal Antibody | Donkey |
| anti-Mouse IgG, Alexa Fluor Plus  555 | AB_2762848 | Donkey anti-Mouse IgG (H+L) Highly Cross-Adsorbed Secondary Antibody, Alexa Fluor Plus 555 | Mouse | **A-32773** | Polyclonal Antibody | Donkey |
| Anti-FLAG-Antibody |  | DYKDDDDK Tag (D6W5B) Rabbit mAb(Binds to same epitope as Sigma's Anti-FLAG M2 Antibody) | Human, Mouse, Rat, Pig,Bovine, Goat, Monkey, Chicken | 14793S | Monoclonal Antibody | Rabbit |
