# Supplementary material for: Sam50 exerts neuroprotection by maintaining the mitochondrial structure during experimental cerebral ischemia/reperfusion injury in rats
Source: CNS Neurosci Ther. 2022 Sep 8;28(12):2230–44. doi: 10.1111/cns.13967 (PMC9627377; doi:10.1111/cns.13967)

Full unedited gel/blot for Figure 1F

NO.1

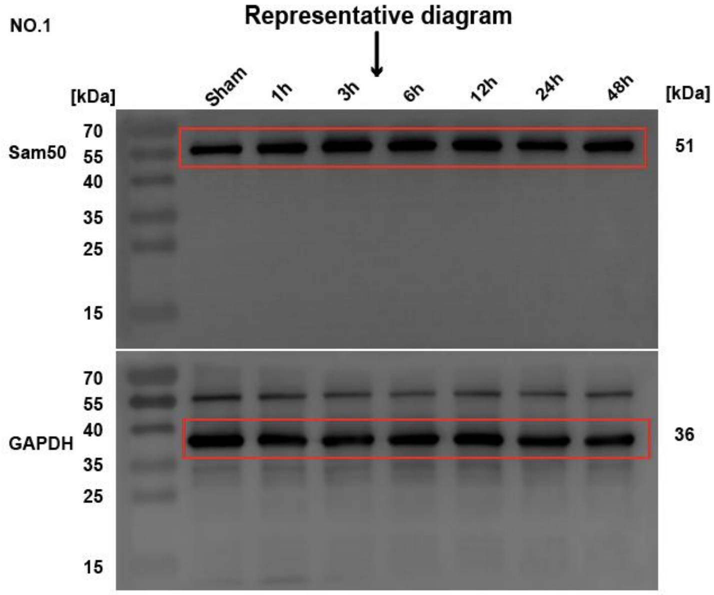

NO.2

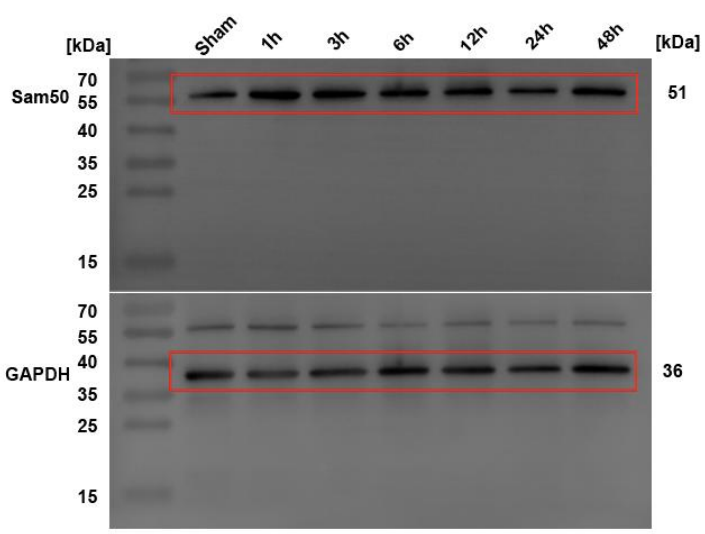

NO.3

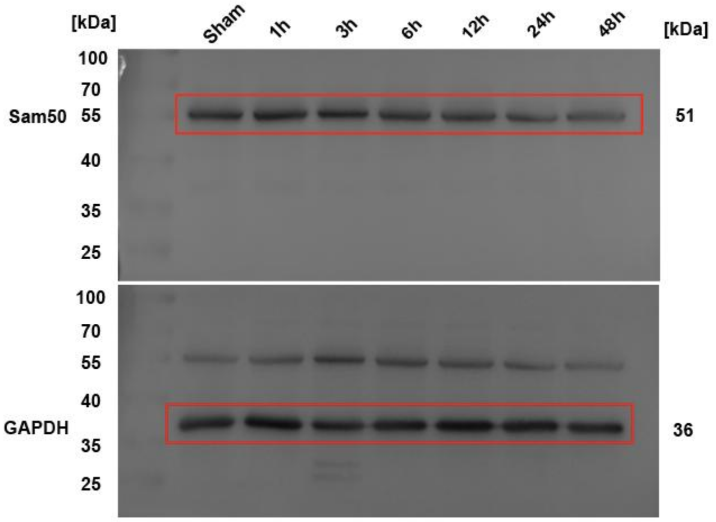

NO.4

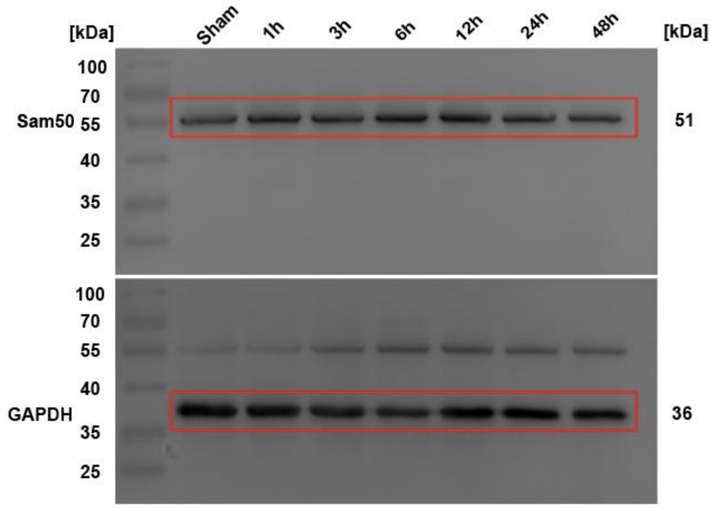

NO.5

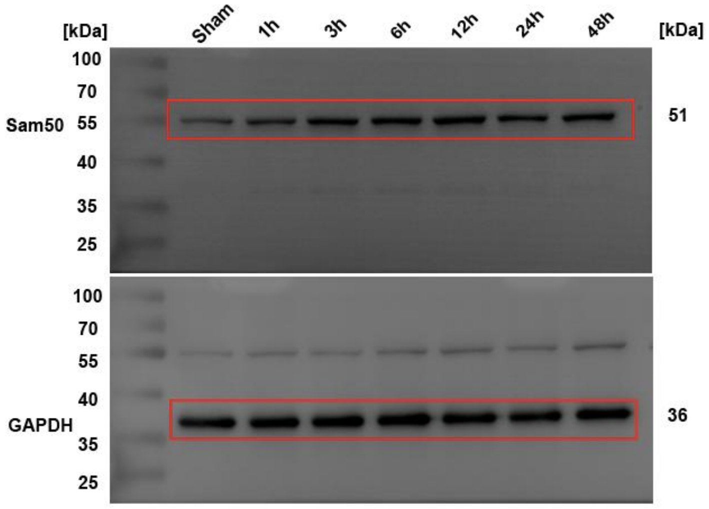

NO.6

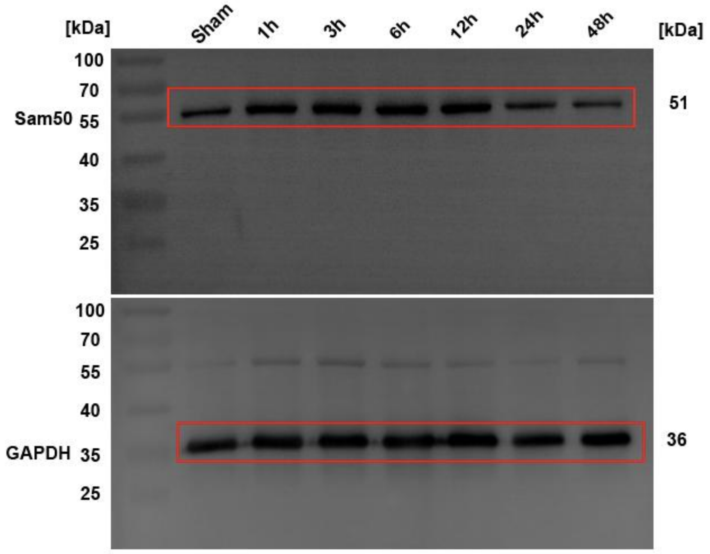

Full unedited gel/blot for Figure 2B

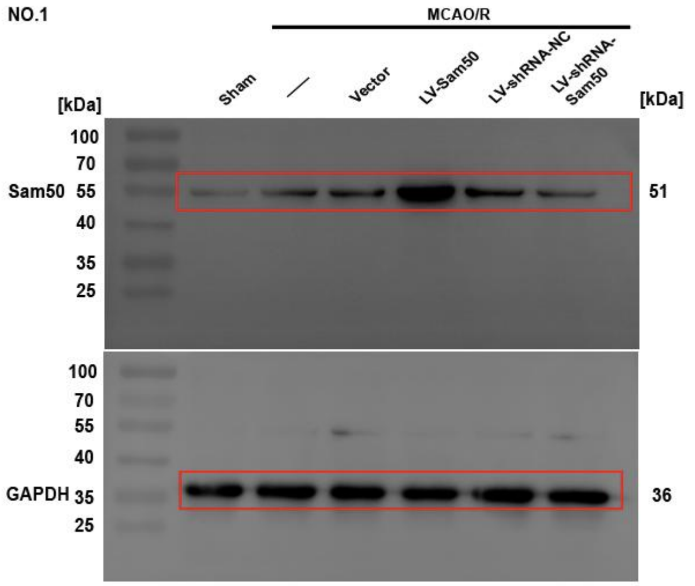

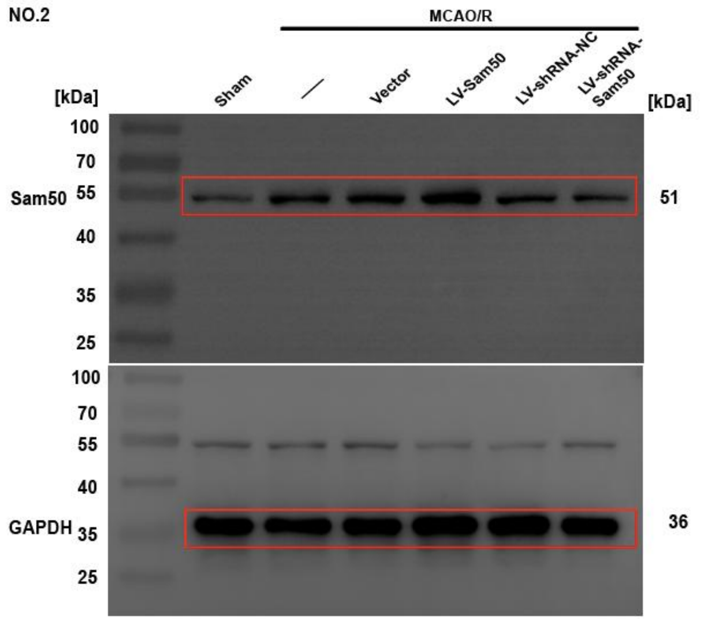

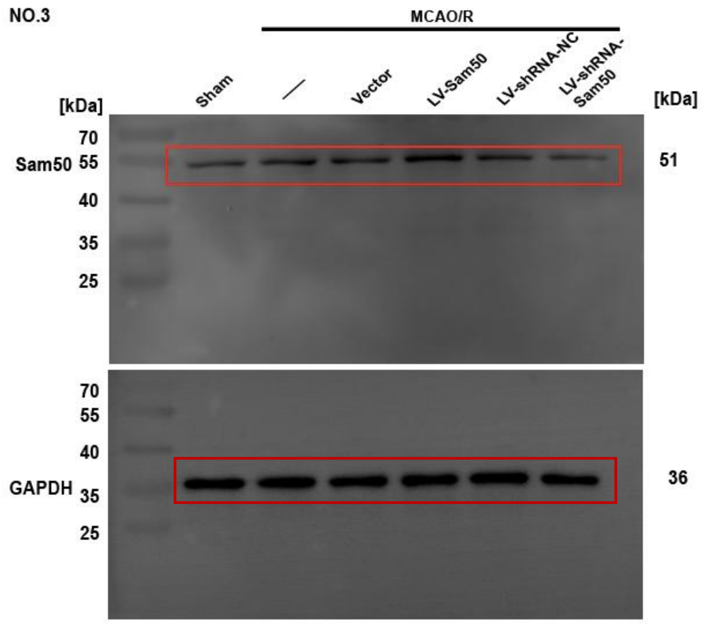

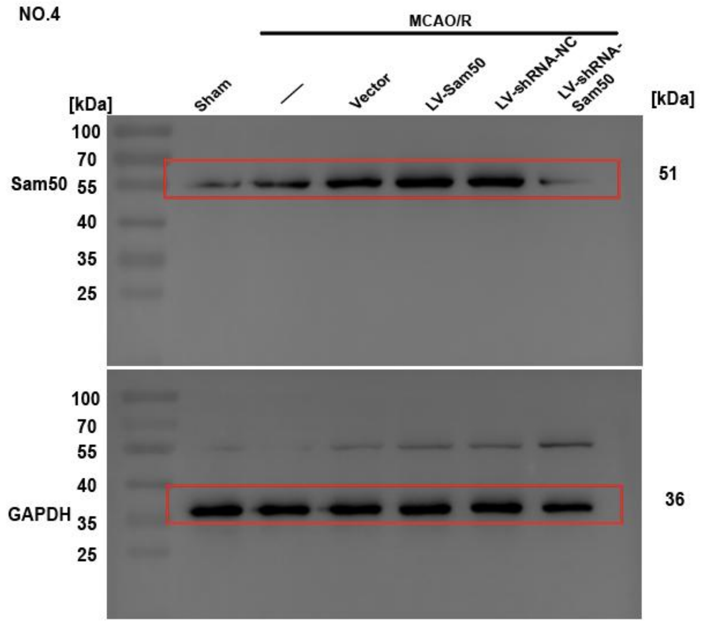

Representative diagram

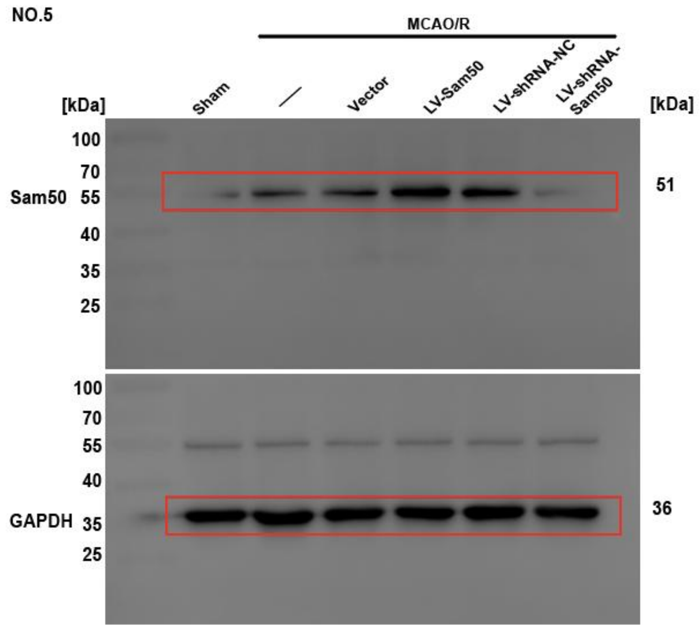

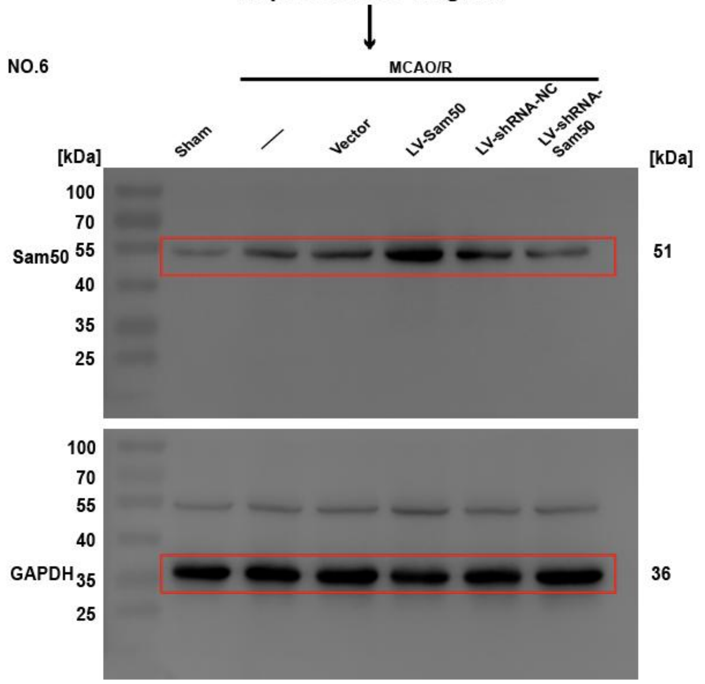

Full unedited gel/blot for Figure 5B

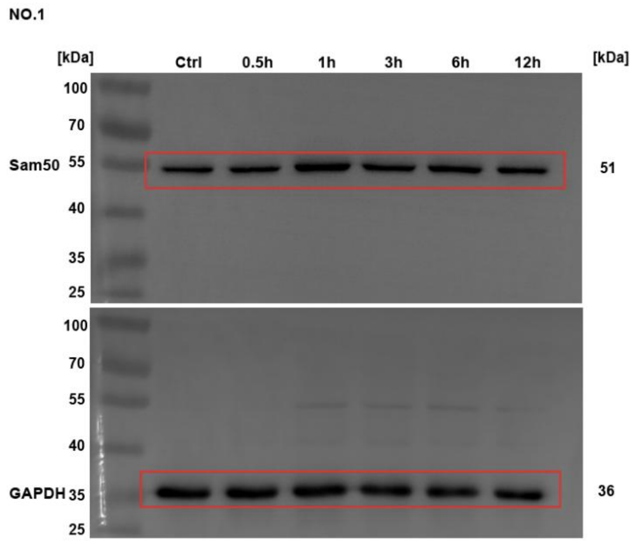

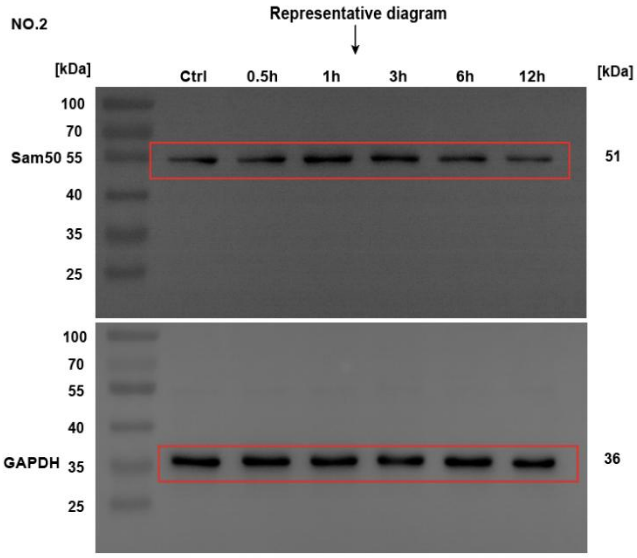

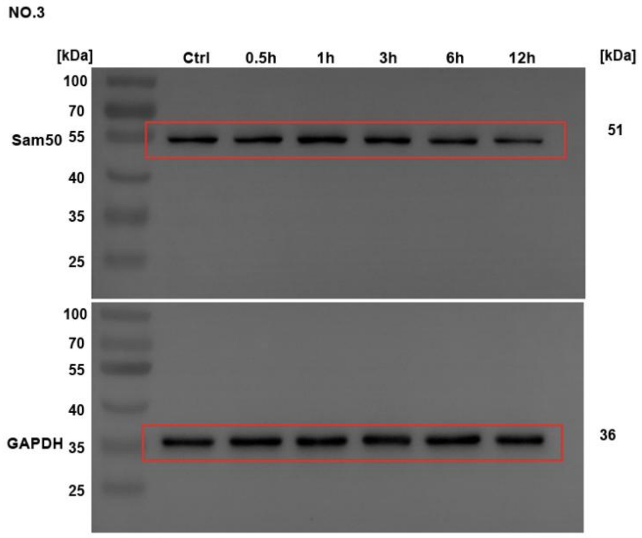

Full unedited gel/blot for Figure 5D

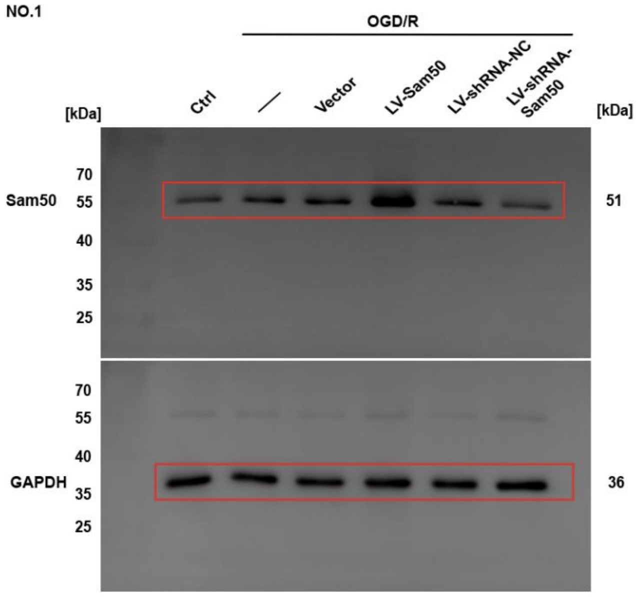

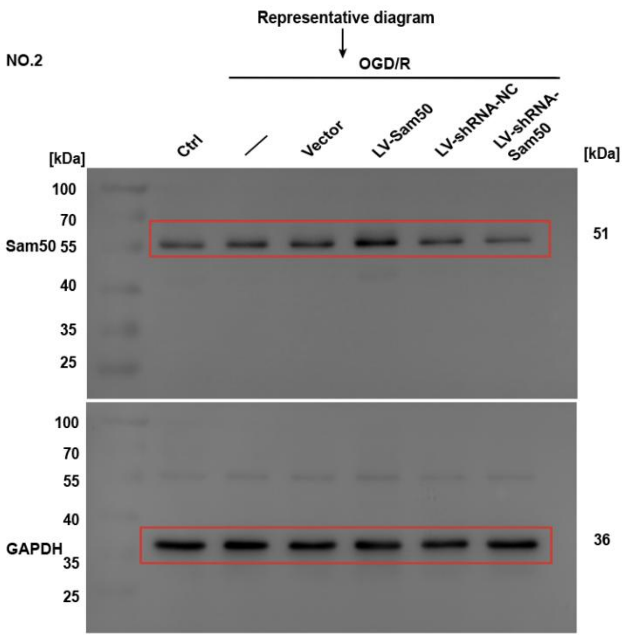

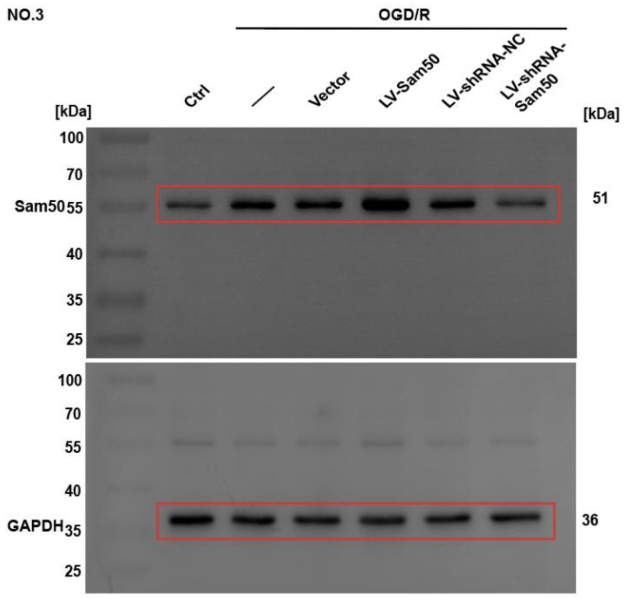

Full unedited gel/blot for Figure 6F

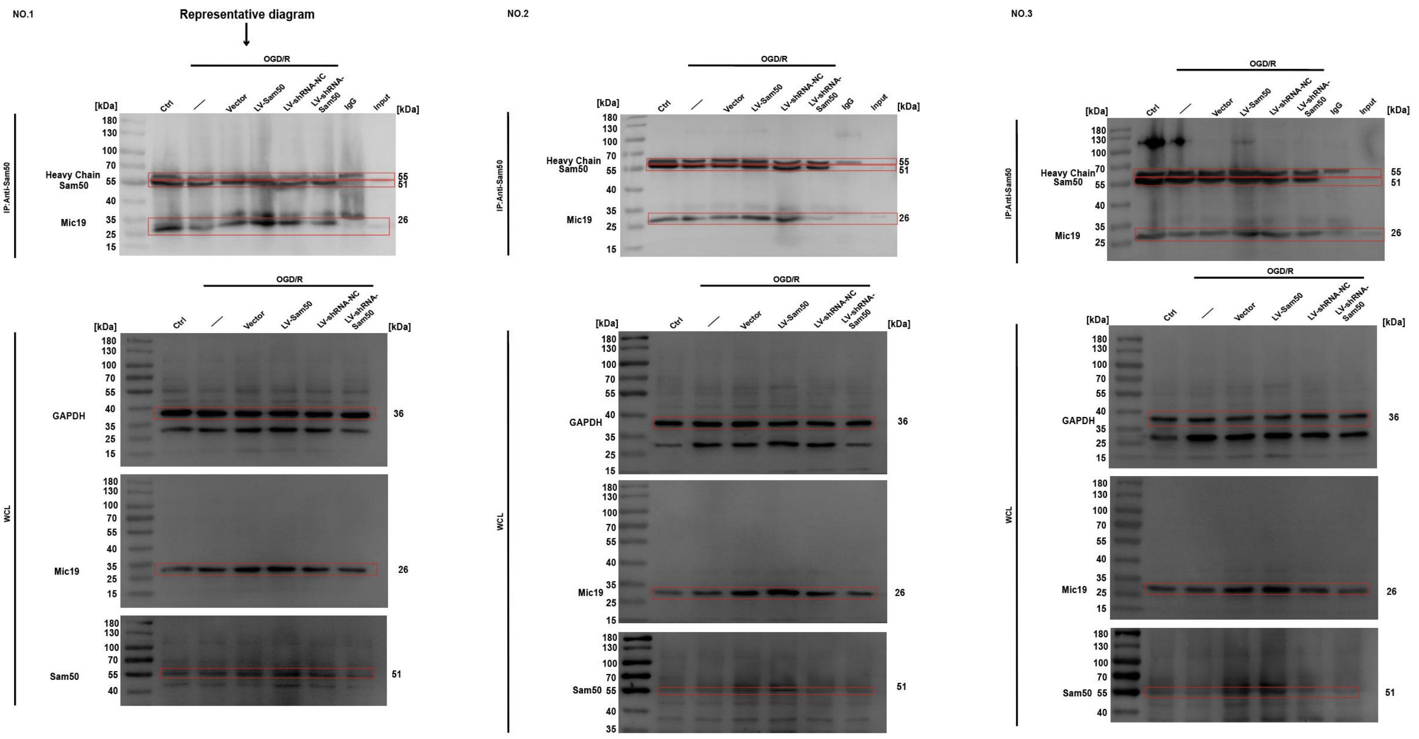

Supplement: Supplementary file 2 — Appendix S2 [file CNS-28-2230-s001.pdf]
